# Widespread sharing of pneumococcal strains in a rural African setting: proximate villages are more likely to share similar strains that are carried at multiple timepoints

**DOI:** 10.1099/mgen.0.000732

**Published:** 2022-02-03

**Authors:** Madikay Senghore, Chrispin Chaguza, Ebrima Bojang, Peggy-Estelle Tientcheu, Rowan E. Bancroft, Stephanie W. Lo, Rebecca A. Gladstone, Lesley McGee, Archibald Worwui, Ebenezer Foster-Nyarko, Fatima Ceesay, Catherine Bi Okoi, Keith P. Klugman, Robert F. Breiman, Stephen D. Bentley, Richard Adegbola, Martin Antonio, William P. Hanage, Brenda A. Kwambana-Adams

**Affiliations:** ^1^​ WHO Regional Reference Laboratory (RRL), West Africa Strategy and Partnership, Medical Research Council Unit the Gambia at the London School of Hygiene and Tropical Medicine, Atlantic Road, Fajara, The Gambia; ^2^​ Center for Communicable Disease Dynamics, Department of Epidemiology, Harvard TH Chan School of Public Health, 677 Huntington Ave, Boston, MA 02115, USA; ^3^​ Infection Genomics, Wellcome Sanger Institute, Hinxton, UK; ^4^​ Darwin College, University of Cambridge, Silver Street, Cambridge, UK; ^5^​ Department of Clinical Infection, Microbiology and Immunology, Institute of Infection and Global Health, University of Liverpool, Liverpool, UK; ^6^​ Respiratory Diseases Branch, Centers for Disease Control and Prevention, Atlanta, GA, USA; ^7^​ Rollins School Public Health, Emory University, Atlanta, USA; ^8^​ Hubert Department of Global Health, Rollins School of Public Health, Emory University, Atlanta, GA 30322, USA; ^9^​ Immunisation and Global Health Consulting, RAMBICON, Lagos, Nigeria; ^10^​ Microbiology and Infection Unit, Warwick Medical School, University of Warwick, Coventry, UK; ^11^​ NIHR Global Health Research Unit on Mucosal Pathogens, Division of Infection and Immunity, University College London, London, UK

**Keywords:** pneumococcal transmission dynamics, rural African setting

## Abstract

The transmission dynamics of *Streptococcus pneumoniae* in sub-Saharan Africa are poorly understood due to a lack of adequate epidemiological and genomic data. Here we leverage a longitudinal cohort from 21 neighbouring villages in rural Africa to study how closely related strains of *S. pneumoniae* are shared among infants. We analysed 1074 pneumococcal genomes isolated from 102 infants from 21 villages. Strains were designated for unique serotype and sequence-type combinations, and we arbitrarily defined strain sharing where the pairwise genetic distance between strains could be accounted for by the mean within host intra-strain diversity. We used non-parametric statistical tests to assess the role of spatial distance and prolonged carriage on strain sharing using a logistic regression model. We recorded 458 carriage episodes including 318 (69.4 %) where the carried strain was shared with at least one other infant. The odds of strain sharing varied significantly across villages (χ^2^=47.5, df=21, *P*-value <0.001). Infants in close proximity to each other were more likely to be involved in strain sharing, but we also show a considerable amount of strain sharing across longer distances. Close geographic proximity (<5 km) between shared strains was associated with a significantly lower pairwise SNP distance compared to strains shared over longer distances (*P*-value <0.005). Sustained carriage of a shared strain among the infants was significantly more likely to occur if they resided in villages within a 5 km radius of each other (*P*-value <0.005, OR 3.7). Conversely, where both infants were transiently colonized by the shared strain, they were more likely to reside in villages separated by over 15 km (*P*-value <0.05, OR 1.5). PCV7 serotypes were rare (13.5 %) and were significantly less likely to be shared (*P*-value <0.001, OR −1.07). Strain sharing was more likely to occur over short geographical distances, especially where accompanied by sustained colonization. Our results show that strain sharing is a useful proxy for studying transmission dynamics in an under-sampled population with limited genomic data. This article contains data hosted by Microreact.

## Data Summary

The sequence reads for the project were submitted to the European Nucleotide Archives. The publicly available accession numbers are available in Table S1, available in the online version of this article. The phylogenetic trees for prevalent serotypes can be visualized in microreact using the following links:

23B: https://microreact.org/project/qReinitt6K1wps7XUt1AGw

15A: https://microreact.org/project/3a2MBXXNx1RaWyiSG9RdCm

6B: https://microreact.org/project/7MTTQLnFcRFmQVcNbwWhTy

15B/C: https://microreact.org/project/n1r6KQmOb

6A: https://microreact.org/project/uPYIn9Lit

19A: https://microreact.org/project/WG653y0Px

**Impact Statement**

African children bear the brunt of serious infections caused by *Streptococcus pneumoniae* (the pneumococcus). Nasopharyngeal carriage of the pneumococcus is a prerequisite for disease and onward transmission across individuals via respiratory droplets. However, transmission studies are hampered by the lack of clinical datasets that capture the breadth of diversity in pneumococcal populations and gross under-sampling of transmission networks. Here we utilized a longitudinal birth cohort to gain insights into the transmission dynamics and determinants of strain sharing of the pneumococcus among Gambian infants from a rural setting. We show a higher likelihood of sharing genetically similar strains among infants whom lived within 5 km of each other. Sustained carriage was also associated with living in close proximity. This study adds to our understanding of the potential importance of spatial proximity for pneumococcal transmission among African infants, which may guide future interventions to improve control of pneumococcal disease.

## Introduction

*Streptococcus pneumoniae,* commonly known as the pneumococcus, is a human-adapted commensal that colonizes the mucosal surfaces of the upper respiratory tract, and is a globally important cause of morbidity and mortality. Colonizing the upper respiratory tract is the precursor for invasive pneumococcal disease and the natural state for transmission, making it a crucial component of the pneumococcal life-cycle [1]. The high rates of pneumococcal carriage in The Gambia, where over 90 % of infants are colonized at least once within the first year of life, make it an ideal setting to study the dynamics and key determinants modulating the spread of pneumococcal strains in the population [2–4]. Prior to the introduction of the 7-valent pneumococcal conjugate vaccine (PCV7) in The Gambia in 2009, the non-random distribution of multi-locus sequence types (MLSTs) within households in Sibanor, in the Western Region of The Gambia, was associated with ongoing transmission of pneumococcal strains [4]. However, important questions remain unanswered regarding the frequency with which strains are shared within communities, partly because resolving transmission routes is complex and requires robust well-sampled datasets.

Transmission across hosts allows bacterial strains to proliferate and spread in a population [5, 6]. Advances in genomics, the integration of epidemiological techniques like contact tracing and applications of mathematical models are increasingly empowering scientists to identify cases that are linked by direct transmission and to infer who infected whom [7–15]. However, understanding transmission dynamics in low-resource settings like The Gambia, where infectious diseases are endemic, remains challenging due to the lack of genomic datasets and epidemiological surveillance data that can be leveraged to delineate transmission chains [16, 17]. Existing genomic datasets from low-resource settings are typically sequenced retrospectively from studies that were not designed to study transmission. Additionally, the lack of genomic datasets from longitudinal studies has thus far hampered our ability to investigate the role of within-host diversity, which can be a useful tool for setting thresholds for strain sharing or recent transmission. In order to better understand transmission, innovative methods, which are not excessively compromised by sampling biases, need to be applied.

An alternative approach is to quantify the frequency with which a given strain is shared by two hosts and use co-variates on host demography and epidemiology to identify the key determinants of strain sharing. Croucher *et al*. pioneered this approach by using a Bayesian framework to stratify the pneumococcal population structure into sequence clusters and computing pairwise SNP difference between strains in the same sequence cluster. Over a range of SNP thresholds, they determined the proportion of pairwise comparisons involving hosts from the same geographic location, and showed that closely related strains were increasingly more likely to be from the same geographic location [18]. Using the same general framework, Chan *et al*. showed that patients were more likely to share near-identical strains of methicillin-resistant *Staphylococcus aureus* if they were admitted to the same hospital. They also showed that inter-hospital sharing of near identical strains increased with patient sharing between hospitals [19].

Here we present insights into the dynamics of pneumococcal strain sharing among regularly sampled infants residing in villages in rural Gambia. We analysed 1074 pneumococcal genomes isolated from infants in an intensively sampled longitudinal birth cohort [20]. We computed pairwise genetic distances between isolates belonging to the same global pneumococcal sequence cluster (GPSC) and defined strains based on serotype and MLST combinations. The mean within-host diversity was calculated for each strain that colonized an infant at multiple timepoints and this was averaged across the dataset to provide an arbitrary threshold for studying strain sharing. This enabled us to define strain sharing where the same strain was carried by two infants and the pairwise genetic distance could be accounted for by the mean within-host intra-strain genetic diversity. Despite only sampling a few infants per village (on average four) and not sampling older contacts, we show evidence of strain sharing across the region and that geographic proximity enriches for strain sharing, especially where accompanied by sustained colonization. This simple approach provides a basis for understanding the determinants of the transmission dynamics and the force of infection [21], even though the population is not widely sampled.

## Methods

### Study design

The genomes analysed in our study were collected as part of the Sibanor Nasopharyngeal Microbiome (SNM) study, which collected 1595 nasopharyngeal swab (NPS) specimens from 102 infants (an average of just under 16 swabs per infant) (Table S2) [20]. Infants were recruited from 21 villages (Table S3) covering an area of 95 km^2^ in the Western Region of The Gambia between November 2008 and April 2009 (a maximum of ten infants were recruited per village) as part of a cluster randomized trial where villages were stratified into three groups. Group 1 consisted of infants from unvaccinated communities where infants received PCV7 after 6 months, in groups 2 and 3, study participants received three doses of PCV7 at 2, 4 and 6 months, but were from unvaccinated and vaccinated communities, respectively. The number of infants sampled in each village ranged from 1 to 10 (mean 4.7, median 4). NPSs were collected from the infants bi-weekly from birth to 6 months and then bi-monthly up until their first birthday. Pneumococcus was cultured and identified in 1258/1595(78.9 %) NPS specimens using conventional microbiological techniques [20].

### Microbiology, DNA extraction and whole genome sequencing

The collection of nasopharyngeal swabs and isolation of pneumococcal colonies were done using standard bacteriologic protocols as previously described [20]. DNA was extracted from pure fresh overnight cultures of the pneumococcal strains as described elsewhere [22]. The isolates were sequenced on the Illumina HiSeq 4000 platform to produce 100 bp paired-end reads as part of the Global Pneumococcal Sequencing project (GPS) (www.pneumogen.net) [23]. The genome data was deposited in the European Nucleotide Archive (Appendix 2).

### Initial handling of paired-end Illumina sequence reads

A total of 1074 genomes were analysed after passing The Sanger Institute’s quality control, which included mapping sequenced reads to the pneumococcal reference genome (accession number FM211187; ST81, serotype 23F) and robust *de novo* genome assembly using an in-house pipeline developed by the Sanger institute [24, 25]. Samples were only included when the overall sequencing depth was at least ≥20×, reads mapping to ≥60 % of the bases in the reference genome, the assembly length was between 1.9 and 2.3 Mb with no more than 500 contigs, and the number of assembled contigs was ≤500. Additionally, samples suspected to compose a mixture of two or more pneumococcal strains were excluded if 15 % or more of the variable sites represented heterozygous SNP sites (over the total SNP sites and >25 % maximum minor allele frequency) were suggestive of a mixture of two or more pneumococcal strains in one DNA sample and were thus excluded. All assembled genomes were annotated using Prokka (1.14.5) [26]. PneumoCaT (Pneumococcal Capsule Typing; https://github.com/phe-bioinformatics/PneumoCaT), a whole-genome-based serotyping method was used to infer serotype *in silico*. MLST types (STs) were inferred by comparing *de novo* contigs against the PubMLST database using blast [24].

### Genomic pairwise distance analysis

To mitigate the effects of potential phylogenetic bias we employed a reference free method for computing phylogenetic distances between isolates [23]. Population Partitioning Using Nucleotide K-mers (PopPUNK) was used to assign strains to Global Pneumococcal Sequence Clusters (GPSCs), based on the international genomic definition of pneumococcal lineages [25]. For all isolates within a given GPSC, we generated an alignment of core genes using Roary (3.12.0) [27], which was parsed through SNP-sites to generate an alignment of variable sites from the core-gene alignment [28]. Within each GPSC we computed pairwise genetic differences for all isolates by counting the number of variable sites using snp-dist (https://github.com/tseemann/snp-dists). The pairwise SNP distances across all GPSCs were amalgamated into one spreadsheet and annotated with metadata including village of origin, date of collection, strain serotype and sequence type.

### Statistical analysis

Statistical analysis was carried out in R and Graphpad Prism (8.3.0). The R package Vegan (V 2.5–6) was used to calculate the Shannon Alpha diversity index for each village, based on the number of unique strains in each village as [−Σ log(*b*)*p_j_*] where *p*_*j*_ is the proportional abundance of strain *j* and *b* is the base of the logarithm. We used the Haversine formula in the R package geosphere (V 1.5–10) to calculate the spatial distance between villages based on their GPS coordinates. The unequal numbers of infants sampled across villages precluded our ability to perform a robust pairwise comparison between villages. Instead, we aggregated spatial distance into three categories: short distance (0–5 km), medium distance (5–15 km) and long distance (15 km or more), and studied the dynamics of strain sharing within these three spatial distance radii. We approximated that <5 km is a walkable distance, 5–15 km is feasible on donkey cart/bicycle distance and longer than that most people would need to use a motor vehicle. At regular SNP thresholds from 0 to 500 SNPs (0–100 SNPs interval size=5 SNPs, 100–250 interval size=10 SNPs, 250–500 interval size=50), we calculated the proportion of comparisons within each spatial distance range [*N*_*ki*_ /*N*_*i*_] where *N*_*ki*_ is the number of comparisons in distance range *k* with *i* or fewer SNPs and *N*_*i*_ is the total number of comparisons within SNP threshold *i*. This value was normalized by the total number of comparisons in each distance range *k* (*N*_*k*_) and the total number of comparisons (*N*):

$$\pi =\frac{N_{Ki}*N}{N_{i}*N_{k}}$$

The change in normalized proportion (π) plotted against the number of pairwise SNPs using the *geom_smooth* function in ggplot2.

### Strain sharing analysis

To investigate the extent to which the degree of genetic relatedness could be considered strain sharing, we computed within host diversity: the SNP distance table was filtered to only include comparisons where the same strain was recovered from the same infant at different timepoints. We defined strains based on the serotype and ST, and to avoid redundancy, we chose a random isolate of each strain in each infant, which yielded a representative subset of 458 genomes. We defined strains as shared if the isolates had a pairwise SNP distance of 50 SNPs or less in the representative subset. In total, 50 SNPs were set arbitrarily to accommodate a mean within host pairwise distance of 23 SNPs in both hosts.

We categorized each strain as a transient or sustained colonizer based on how many times the strain was isolated from the infant; if the strain was isolated at three or more visits it was defined as sustained colonization and if it was only isolated once or twice it was defined as transient colonization, regardless of whether they were isolated on successive visits. Each pairwise comparison was categorized into one of three groups depending on the nature of the colonization in each child:

1. Sustained–sustained: sustained colonization in both infants;
2. Sustained–transient: sustained in one infant and transient in the other;
3. Transient–transient: shared strain was transiently colonized both infants.

A Chi-squared test was implemented to test for a difference in the distribution of sustained, mixed and transient pairs across spatial distances. Differences in the distribution of mean pairwise distance across the three categories were tested statistically using a Mann–Whitney U test. To ensure that the pairwise distance was not a random artefact, for each shared strain each, we did an all vs all pairwise comparison of all isolates of the strain vs all isolates of the same strain in the second infant and computed the mean pairwise distance between the two infants.

### Logistic regression for strain sharing and village network analysis

We implemented a generalized linear logistic regression model to test for an association between an infant residing in a given village and the odds of a strain being shared. Our binary outcome was whether or not the strain was shared and we computed the odds ratios for strain sharing in each village while adjusting for whether strains were PCV7 serotypes and how many times the strain was isolated in the infant. A similar analysis was done to study potential associations between global lineages and strain sharing.

### Phylogenetic analysis

For the most prevalent serotypes a reference from the same or closely related serotype was chosen. Sequencing reads were mapped to the reference by the Burrows Wheeler Aligner (BWA) and variable sites were called based on at least five reads mapping to the site and at least 75 % agreement among reads. A consensus sequence was generated using samtools and all the consensus sequences were concatenated into a multifasta alignment. The multifasta alignment was inputted in Gubbins [29], which identified and excluded recombination regions to reconstruct a high-resolution phylogeny. The phylogenetic trees were visualized in Microreact with accompanying metadata [30]. To study character states, variable sites were extracted for closely-related strains and were visualized in Seaview version 4 [31].

## Results

### The probability of identifying similar strains increased over shorter spatial distances

**Fig. 1.**
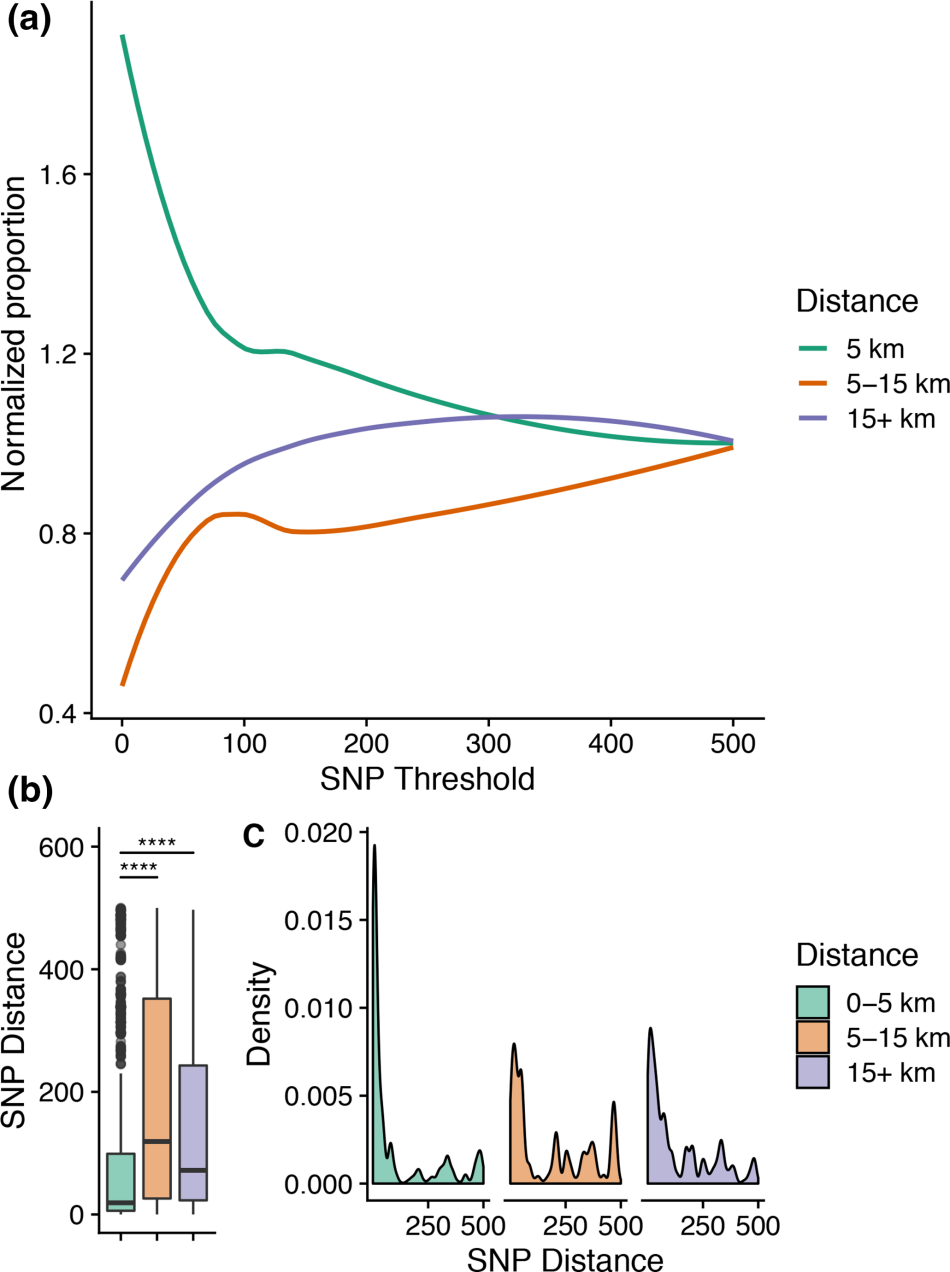
Relationship between pairwise genetic distance of colonizing strains and spatial distance between villages where infants reside. (a) The plot shows normalized proportion of pairwise comparisons within a given spatial proximity range (three categories:0–5 km, 5–15 km and 15+km) below a SNP threshold. (b) Distribution of pairwise SNP distances across the three spatial distance ranges. (c) Frequency density distribution of pairwise SNP distances by spatial distance range. (****=*P*-value <0.0001).

We identified 71 GPSCs and the most common were GPSC44 (79, 7.4 %), GPSC51 (71, 6.6 %), GPSC60 (66, 6.1 %), GPSC26 (65, 6.1 %), GPSC75 (51, 4.7 %), GPSC81 (50, 4.7 %) and GPSC25 (48, 4.5 %). We showed that a greater proportion of closely related strains were among infants from proximal villages (within 5 km of each other) (Fig. 1a). Moreover, comparisons involving infants from the same village or proximal villages had a significantly lower pairwise genetic distance compared to strains recovered over longer distances (Mann–Whitney U-test, *P*-value <0.005) (Fig. 1b, c). We went further to define strain sharing as a proxy for understanding the patterns of transmission in the community. We classified strain sharing based on an arbitrary SNP threshold of 50 SNPs to accommodate the mean within-host variation among both infants (mean within host diversity 23 SNPs, median 6 SNPs). To avoid redundancy, we chose a random variant of each strain in each infant, which yielded a representative subset of 458 genomes. Among these, 318(69.4 %) were shared with at least one other infant. On average, shared strains were seen in three other infants (mean 3.47, median 3), and one strain of serotype 15B/C_ST910 in GPSC 27 was shared by 12 infants.

### Prolonged colonization with shared strains is more likely across proximal villages

**Fig. 2.**
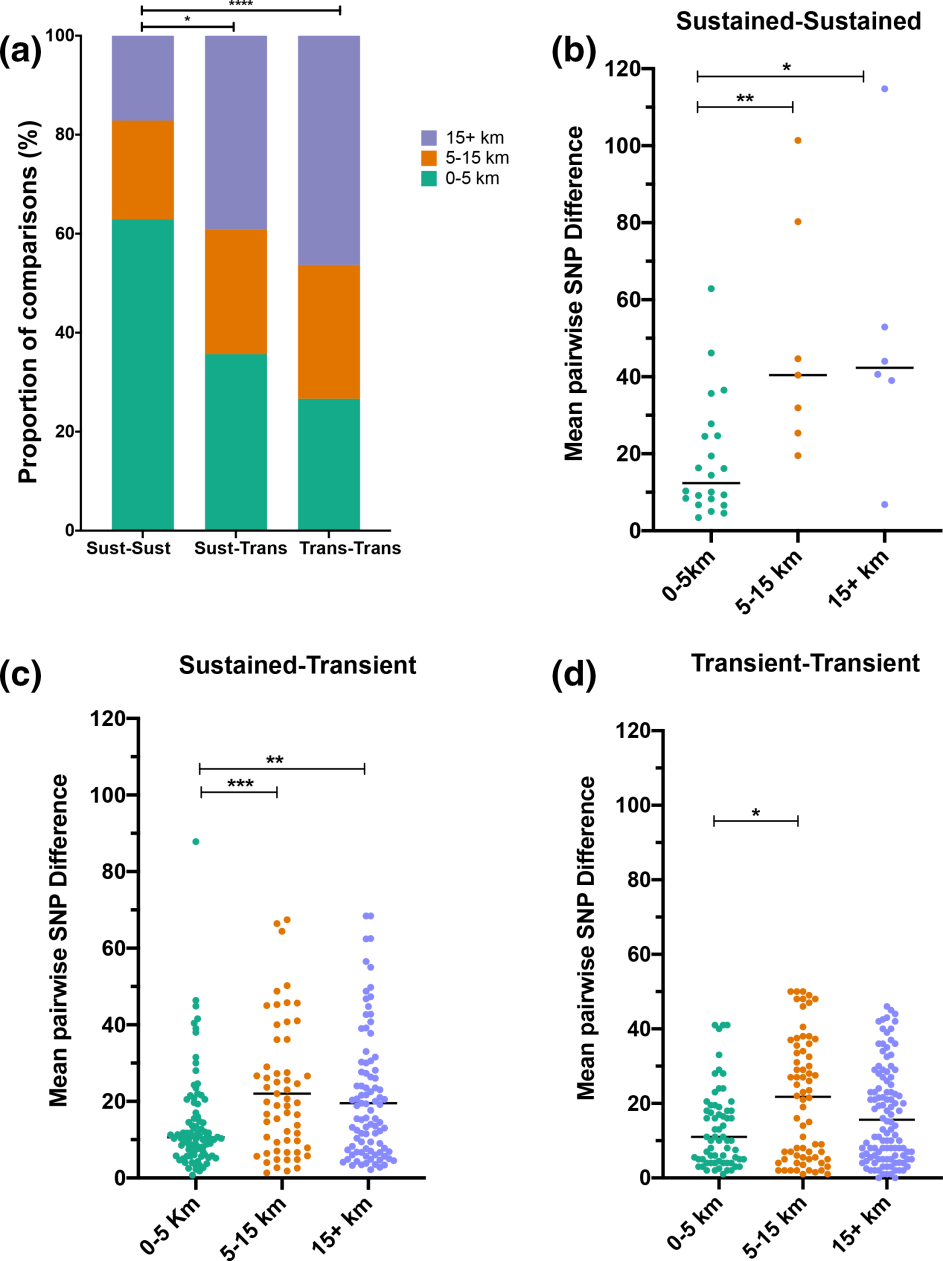
Effect of spatial distance on the dynamics of strain sharing involving infants with sustained colonization, transient colonization or a mixed pair of infants. (a) Spatial distribution of strains shared between infants with sustained carriage of shared strains, transient colonization in both or a mixed pair of two infants with sustained and transient carriage respectively. (b–d) Distribution of mean pairwise SNP differences across all variants of the strain among infant pairs by spatial distance in the three categories: sustained carriers, mixed pairs and transient carriers, respectively. (*P*-value; *<0.05; **<0.005; ****<0.0001).

We then tested whether strain sharing involved pneumococci that transiently colonized infants or were long-term/sustained colonizers, and how this was affected by the spatial distance between the infants. Over half of shared strain pairs involved at least one infant that had sustained colonization: 35 (6.4 %) pairs involved infants that both had sustained colonization with the shared strain, 258 (46.7 %) involved one infant with sustained colonization and another infant that was transiently colonized at one or two timepoints, while 259 (46.9 %) involved two infants that were both transiently colonized by the shared strain. The spatial distribution of infants sharing strains in these three categories varied significantly (Fig. 2a). Infants were significantly more likely to carry a sustained and shared strain if they resided in proximal villages (<5 km radius) (odds ratio 3.742, 95 % CI 1.874–7.596 *P*-value <0.0005). Conversely, if the infants were both transiently colonized by the shared strain they were significantly more likely to reside in villages separated by a long distance (over 15 km) (odds ratio 1.501, 95 % CI 1.061 to 2.098, *P*-value <0.05).

The sustained shared strains were genetically more similar between children residing within a short distance of each other (*P*-value <0.005). Overall the mean pairwise distances between shared strain from all timepoints among infant pairs was 19 SNPs (95 % CI 11.7–25.3, median 12 SNPs) over short distance compared to 49 SNPs over both medium distance (median 40 SNPs, 95 % CI 21.0–77.2) and long distance (median 42 SNPs, 95 % CI 12.4–87.0) (Fig. 2b, c). An example of a strain that was shared and carried for a sustained duration among infants is 19A_ST10542 (GPSC5). This strain colonized numerous infants from neighbouring villages II and SS at multiple timepoints, and transiently colonized two other infants from distant villages (EE and GG) (Fig. S1)

### Villages are densely connected by strain sharing

**Fig. 3.**
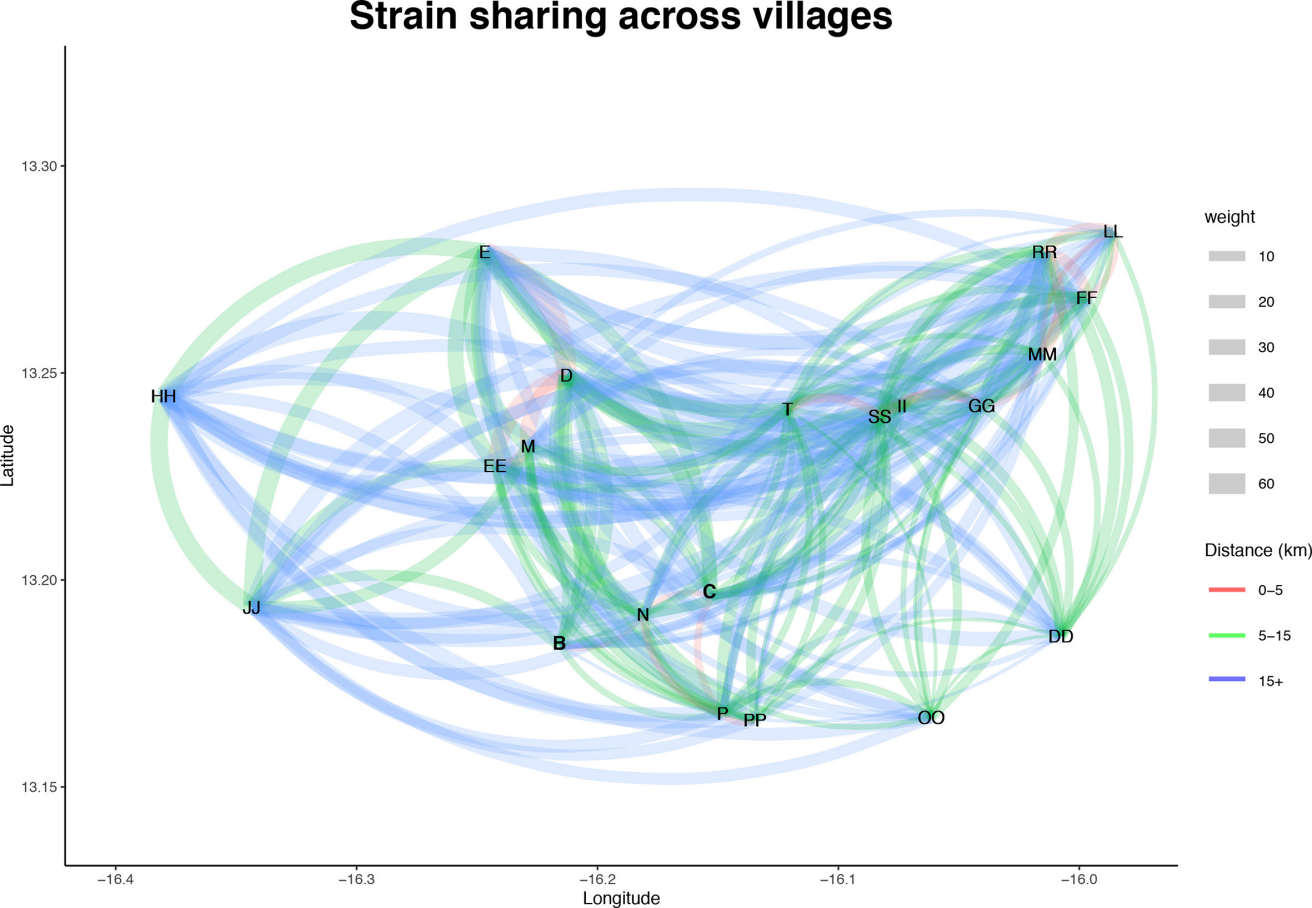
Trends in strain sharing across villages. A network of strain sharing across villages, each node represents a village and villages are plotted based on the GPS latitude and longitude coordinates. Each undirected edge represents strain sharing between the two villages and edges are weighted based on the number of shared strains. Edges are coloured based on the distance between the villages.

We studied the transmission trends between villages by reconstructing a network where nodes were placed based on the GPS coordinates of the village and each edge was weighted by the number of strains shared between two villages. The network shows widespread strain sharing across the study area over proximal, medium and long distances (Fig. 3). The odds of a strain being shared varied significantly based on which village the infant resided (χ^2^=47.5, df=21, *P*-value <0.001).

To identify potential hotspots we ran a logistic regression on a representative subset of 458 genomes, and found that the neighbouring villages D, EE and M were significantly associated with strain sharing (*P*-value <0.1) (Tables S4 and S5). Out of 458 carriage episodes 62 (13.5 %) expressed a PCV7 serotype and 108 (23.6 %) involved other PCV13 serotypes. Strains belonging to PCV7 were significantly less likely to be involved in strain sharing after controlling for village (log OR −1.07, *P*-value <0.001; χ^2^=9.78 df=1, *P*-value=0.002). A potential confounder was that some of the infants were vaccinated early and came from villages with PCV7 exposure. However, there was no significant difference in the odds ratio for sharing across the three arms of the trial, despite differences in vaccine exposure for the infants.

## Discussion

We studied pneumococcal strain sharing in order to understand the transmission dynamics in The Gambia, a high disease burden setting. Despite only sampling a handful of infants per village and not having any isolates from close contacts, we were able to show that children in close proximity to each other were more likely to be involved in strain sharing, but we also show a considerable amount of strain sharing across longer distances. We interpret this as the consequence of rapid transmission and dissemination in this setting underpinned by a high force of infection, which begins to explain why Gambian infants are colonized by the pneumococcus very early in life: colonization rates peak between 2 and 5 months [20, 32]. Pneumococcal transmission is likely enhanced by viral agents, which we now know are important contributors to acute upper respiratory tract infection in The Gambia, as well as the dry season, which is characterized by low humidity and dust [33–35]. Additionally, the communal extended family living arrangements in Gambia increase the infants’ contact rate with other young children and toddlers, who are the principal drivers of pneumococcal transmission [36, 37].

The longitudinal sampling in this dataset allowed us to go further and determine whether shared strains were being carried at multiple timepoints or only transiently carried, and how this was related to village proximity. Our dataset included instances where infants were only transiently colonized by a given strain as well as others where a strain was carried and sustained over multiple time points (three or more). This was not surprising since both transient and persistent pneumococcal carriage have been reported in infancy over durations that vary across serotypes [36, 38]. Where we noted strain sharing between a pair of infants, we were more likely to observe sustained carriage in both infants if they resided within close geographic proximity, whereas a longer distance separation favoured transient colonization by the shared strain in both infants. In order to sustain carriage in an infant, a strain must also overcome competition with other microbes within the host and evade the host immune system [39–41]. Given that proximity enhances sustained carriage by a shared strain and that infants are colonized by multiple strains over the course of the year, there may be a role for repeated exposure in the sustained carriage of a shared strain. However, this raises important questions because although we know that concomitant carriage of multiple strains is possible, it is unclear how many pneumococcal strains can co-exist in the upper respiratory tract at a given time and for how long they can co-exist [40, 42]. Microarray serotyping data from Malawian children showed that 44 % of carriage events had between two and six pneumococcal serotypes [43]. Furthermore, it is not clear whether infants develop immunity to colonizing strains and how this can affect their susceptibility to recolonization with the same strain.

Given the lack of genomic datasets powered to infer routes of transmission, our approach to study strain sharing in a longitudinal intensively sampled population has provided insights into pneumococcal transmission in The Gambia and its potential determinants. Carriage rates remain high and pneumococcus remains a major cause of morbidity and mortality in The Gambia, prompting the need to explore novel strategies to curb the burden of disease [2, 3, 44, 45]. Pneumococcal conjugate vaccines are currently the most effective means of lowering the burden of pneumococcal carriage and disease. In our study, PCV7 serotypes were significantly less likely to be involved in strain sharing, suggesting an indirect effect due to study participants and some communities being vaccinated. However, the overall rates of strain sharing did not vary significantly between communities that were exposed to vaccination compared to unvaccinated communities. This may be linked to the fact that conjugate vaccines only target a limited number of serotypes (currently up to 13) and the benefits of eliminating vaccine serotypes can be undermined by the increased prevalence of non-vaccine serotypes and non-typeable strains [20, 46, 47], which may lead to replacement disease. Our previous analysis showed that in carriage, PCV7 serotypes were immediately replaced by non-PCV7 serotypes following vaccination [20]. Reductions in the incidence of invasive disease caused by the pneumococcus and other respiratory pathogens associated with the restrictions and other measures rolled during the CoVID-19 pandemic may provide insights into non-vaccine-based control strategies [48]. Interventions to disrupt overall pneumococcal transmission may be necessary to complement ongoing vaccination efforts, but this requires further understanding of the transmission dynamics in our setting.

Finally, our data suggests that transmission is likely widespread in The Gambia and occurs more frequently between proximal villages. However, further evidence is needed to enable us to confidently model the spread of pneumococcal strains through space and time. Future work, beyond the scope of this project could combine pathogen genomics with human mobility data by partnering with Google, social media or mobile phone companies, to model the expected spread of pneumococcal strains between communities [49]. These approaches may be particularly useful in the use of carriage surveys to predict the trajectory of pneumococcal meningitis outbreaks that occur periodically in the African meningitis belt [22, 50]. Another interesting relationship that warrants further investigation is the relationship between the transmissibility of pneumococcal strains and virulence [5, 6]. Opportunistic pathogens like the pneumococcus challenge the dogma of standard virulence evolution theory that increased virulence is naturally selected for and therefore virulent strains are more likely to transmit and proliferate [51]. Studies of virulence evolution in the pneumococcus have focused on invasiveness and the role of known virulence factors [52–54]. Going forward it will be useful to design studies that encompass contemporaneous carriage and invasive disease datasets so we can assess the relationship between the transmissibility of strains and their propensity to cause invasive disease.

## Study limitations

The main limitation of this study was that it was not designed to study transmission, therefore important links in transmission chains are likely to have been missed. When we set an arbitrary threshold, we did not account for variable clock rates in different lineages. A further limitation is that we only picked one colony per NPS specimen, we probably underestimate strain sharing because we did not pick multiple colonies. We also have not accounted for time and age of the infants.

## Supplementary Data

Supplementary material 1

Supplementary material 2
