# Supplementary material for: Widespread sharing of pneumococcal strains in a rural African setting: proximate villages are more likely to share similar strains that are carried at multiple timepoints
Source: Microb Genom. 2022 Feb 3;8(2):000732. doi: 10.1099/mgen.0.000732 (PMC8942022; doi:10.1099/mgen.0.000732)

Supplementary Figure 1: The phylogenetic relationship of serotype 19A ST10542 genomes that colonized infants in the first year of life. A) A phylogenetic tree annotated with the village the infant resided at and tips were labelled with the infant's ID and the visit number. B) A timeline for when isolates were recovered annotated by village. C) An alignment showing the nucleotide character states of all non-recombinant variable sites in the core genome.

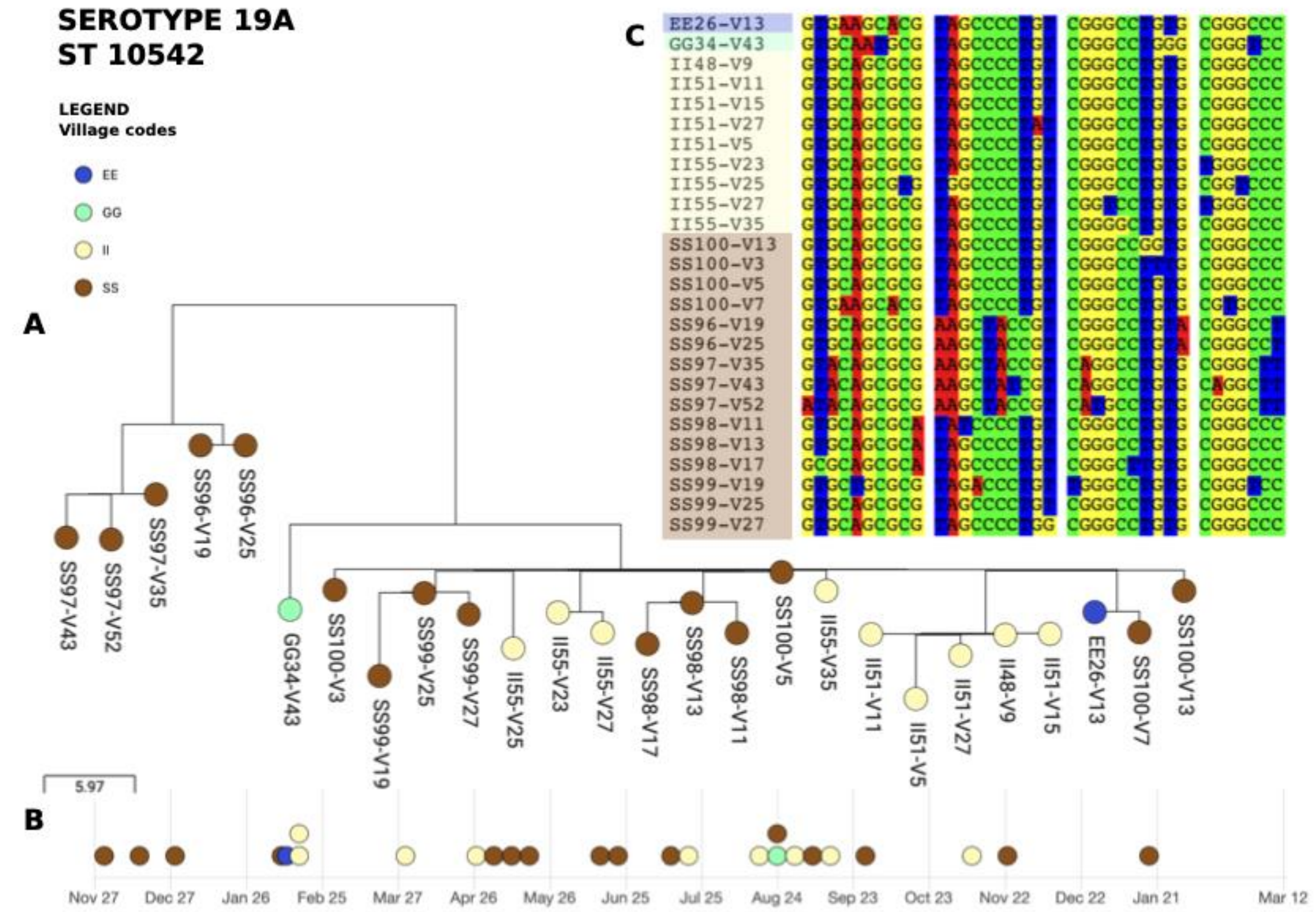

Supplement: Supplementary material 1 [file mgen-8-0732-s001.pdf]
